# Relationship of socioeconomic status, psychosocial factors, and food insecurity with preterm labor: A longitudinal study

**Published:** 2018-09

**Authors:** Mahrokh Dolatian, Nasibeh Sharifi, Zohreh Mahmoodi

**Affiliations:** 1 *Department of Midwifery and Reproductive Health, School of Nursing and Midwifery, Shahid Beheshti University of Medical Sciences, Tehran, Iran.*; 2 *Department of Midwifery, School of Nursing and Midwifery, Ilam University of Medical Sciences, Ilam, Iran.*; 3 *Social Determinants of Health Research Center, Alborz University of Medical Sciences, Karaj, Iran.*

**Keywords:** Socioeconomic status, Food insecurity, Preterm labor, Pregnancy outcome

## Abstract

**Background:**

Premature birth is the main cause of neonatal mortality and long-term complications, which imposes heavy financial and psychological burdens on the family and society; therefore, it is important to recognize the factors affecting it.

**Objective:**

The aim of this study was to determine the relationship between socioeconomic status, psychosocial factors, and food insecurity with preterm delivery.

**Materials and Methods:**

This longitudinal study was conducted on 674 pregnant women at 24-28 wk of gestation who met the inclusion criteria. The subjects were selected using cluster sampling. The pregnant women filled out total questionnaires of study and they followed up until delivery and the data about the newborn was collected after delivery. The data collection tools included questionnaires for evaluating socioeconomic status, psychosocial factors, and food insecurity.

**Results:**

The prevalence of preterm delivery was 7.7%, and socioeconomic factors were not associated with preterm labor. Among the intermediary factors, social health, food insecurity, stress, and prenatal care had a significant relationship with preterm labor. The prevalence rates of preterm delivery in cases with food insecurity, stress, and inadequate prenatal care were 2, 9.1 and 13.2 times higher than those who had food security, did not experience stress, and received adequate care during pregnancy.

**Conclusion:**

Preterm labor is a relatively common problem in which intermediary social determinants of health can play an important role. Considering the limited studies on this issue, the results of this study can lay the foundation for future studies.

## Introduction

The start of a healthy life has been described as a fundamental requirement for each child, and the effects of a good start will remain for a lifetime. Impaired growth can increase physical health risks during life (1, 2). Preterm labor and low birth weight are among the most important indicators for assessing the status of newborns and determining the survival rate in different countries (3). Despite the advances in medical sciences in disease diagnosis and treatment, adverse pregnancy outcomes remain a global problem (4). By definition, preterm delivery refers to births before 37 wk of gestation or less than 259 days from the first day of the last menstruation (5). The prevalence of this outcome is reported to be 9.6%, which is not consistent across different counties. In general, 85% of cases of preterm delivery occur in Asia and Africa, accounting for 3.1% of the global burden of disease (6, 7). In Iran, its prevalence ranges between 5.6% and 34.9%, and in the United States, it is reported to be 12-13% (7, 8).

Different biological (e.g., diseases and malnutrition) and non-biological factors (e.g., social support and domestic violence) affect maternal and neonatal health during pregnancy. Today, health prospect has improved and attention has been focused on the social determinants of health; in recent years, social determinants of health have been considered as the most complex and controversial issues in the area of health policy (9). According to the conceptual framework, Social determinants of health include 4 parts: structural determinants, intermediate determinants, Socioeconomic and political context and the level of health inequity that factors affect one another and ultimately health (2, 7).

As stated, adverse outcomes of pregnancy are influenced by social determinants of health. For example, stressful life events, chronic stressors, pregnancy anxiety, and major events in the society enhance the rate of premature delivery (10). Research has shown that high rate of preterm labor in poor communities may be due to psychosocial and biological factors, and they believe preterm labor is a syndrome that has many causes.

In addition, the results of studies on food insecurity and adverse pregnancy outcomes have highlighted their impacts on maternal outcomes such as gestational diabetes, hypertension, and excessive overweight (11). However, there is a scarcity of studies on the impact of food insecurity on neonatal outcomes such as preterm birth and low birth weight. Given the controversy of the results of studies, the importance and high prevalence of this outcome in pregnancy, and since researchers have not yet determined that whether the cause of this important pregnancy outcome is the interaction of socio-economic, medical, and psychological factors or it can take place as a result of any of these factors independently. 

Thus, this study was designed to determine the relationship between socioeconomic status, psychosocial factors, and food insecurity with preterm delivery.

## Materials and methods

This longitudinal study was conducted on 674 pregnant women at 24-28 wk of gestation meeting the inclusion criteria during April 2016-March 2017 in Ilam Province, Iran. The inclusion criteria were basic literacy, singleton pregnancy, gestational age of 24-28 wk, lack of any known medical conditions, no history of preterm delivery, and consent to participate in the study. 

On the other hand, the exclusion criteria consisted of lack of cooperation with the study and not completing the research questionnaires. Cluster sampling method was used to select the participants. In each city, the number of samples was determined in proportion with the number of women of reproductive age. In each town, a number of urban health centers were randomly selected, and the eligible samples were recruited at intervals of 24-28 wk. For those who met the inclusion criteria, the research objectives were explained and informed consent was obtained from them. The samples were assured that their data would remain confidential and that they were free to leave the study in any time without any effect on their care. 

The participants were interviewed at the entrance to the study, and the questionnaires were completed during 24-28 wk of pregnancy to assess demographic and midwifery data, socioeconomic status, psychosocial factors (stress, anxiety, depression, social support, violence, pregnancy's worries and stress), and food insecurity. The subjects were followed up until delivery to determine the prevalence of preterm labor and pregnancy outcome.

Part of required data were gathered by calling to delivered women and completed through hospital documents. After that the study population were divided in two groups (preterm and term labor)

Sample size was estimated using the formula; 

$n=\frac{1.98^{2}\times 0.155\times (1-0.155)}{0.001^{2}}$

(n=503, p:15.5%; d2:0.001). Considering the sampling method (design effect equal to 1.5) and 10% attrition, the standard sample size was calculated at 834 (7). The data collection tool was a questionnaire consisting of three sections: 1- Demographic and Midwifery questionnaire, 2. Socio-economic status questionnaire, and 3. Questionnaires for identifying the intermediary social determinants of health (i.e., DASS 21, HFIAS, MSPSS, Pregnancy’s Worries and Stress Questionnaire, and Domestic Violence Questionnaire).

Demographic and Midwifery Questionnaire: This questionnaire was designed by the research team and included items on maternal age, age of the spouse, ethnicity, gestational age, gravidity, and intervals between pregnancies, unwanted pregnancy, and the frequency of seeking prenatal care. Socio-economic status questionnaire: This questionnaire was designed by the research team and comprised of items on parental education, parental occupation, household size, household income, and average household costs. Its face and content validities were determined, and its reliability was established using Cronbach's alpha coefficient (α=0.794). The DASS-21 standard questionnaire for stress, anxiety, and depression: This 21-item questionnaire was first presented by Lovibond in 1995, which uses seven questions to measure of the symptoms of stress, anxiety, and depression. This questionnaire is rated using a Likert-type scale (i.e., never, low, moderate, and high). The lowest and highest possible scores for each question are 0 and 3, respectively. This questionnaire has been used in various studies both in Iran and other countries, and its validity and reliability were confirmed (12).

Pregnancy's Worries and Stress Questionnaire: This questionnaire consists of 25 phrases on maternal and neonatal health, personal and family issues, childbirth and the experience of motherhood and mother and newborn preferences.

This questionnaire is rated using a 5-point Likert type scale, with the minimum and maximum possible scores of 0 and 100, respectively. The scores obtained from the questionnaire mark that how worried the pregnant woman is and that which factors are worrying her. Validity and reliability of this scale were approved in Iran (13).

Domestic Violence Questionnaire: This questionnaire was designed by the WHO and measures the violence committed by the spouse during pregnancy, the number of cases of violence calculated based on a 5-point Likert scale. Individuals who give at least one positive response to any of the questions about physical, sexual, or emotional violence are considered to have experienced violence. The validity of the questionnaire was studied in Iran by many researchers and its Cronbach's alpha coefficients were 0.92, 0.89, and 0.88 for the physical, psychological, and sexual violence domains (14).

Multidimensional Perceived Social Support Scale: The Multidimensional Scale of Perceived Social Support (1998) is a 12-item tool developed to evaluate perceived social support from three sources of family, friends, and significant others. The minimum possible score is 12 and the maximum score is 84. The scores of 12-48 indicate low social support, the scores of 49-68 denote moderate social support, and scores 69-84 show high social support. Its validity and reliability were confirmed in Iran; its validity was confirmed through content analysis and its reliability in various studies was established using Cronbach's alpha coefficient (α=0.86-0.9 for the subscales and 0.86 for the whole instrument) (15). 

Household Food Insecurity access scale includes 9 questions with a 4-point Likert scale. This instrument provides information on Household Food Insecurity in terms of access to food. Validation of this questionnaire was previously confirmed by Mohammadi and colleagues (16).

**Ethical consideration**

This article was derived from a doctoral dissertation on reproductive health approved by the Research Committee of Shahid Beheshti University of Medical Sciences (code no sbmu.rec.1394.112). In addition, after explaining the research objectives for the participants, informed written consent was received for participation in the project.

**Statistical analysis**

To analyze the data, descriptive statistics were used to determine frequencies, percentages, means, and standard deviations. Chi-square test, Fisher's exact test, and logistic regression (Because the outcome in study is a two-state (preterm and term labor), Logistic regression were used to determine the effective factors on it and calculate the odds ratio and confidence interval. 

Were run to identify the relationship between the determinants and preterm labor in SPSS software (Statistical Package for the Social Sciences, version 19.0, SPSS Inc, Chicago, Illinois, USA). The statistical tests were performed at the 95% confidence interval.

## Results

Out of the 837 pregnant women, 163 women were excluded from the study due to incomplete questionnaires, intrauterine fetal death, and inaccessible medical records for following up the pregnancy outcomes. The final analysis was performed on the data of 674 subjects. The mean ages of the studied samples and their spouses were 28.59±4.24 and 33.25±5.43 yr, respectively. The mean number of years of education was 13.03±3.28 yr. 

The lowest and highest numbers of gravidities were 1 and 7, respectively. Most of the samples (86.4%) were housewives and lived in households consisting of 1 to 3 peoples (78.2%). Household income in 340 (50.4%) of the population was ten million Rials or more and average household expenditure in 558 (82.8) of the participants was ten million Rials or more. (Table I). In addition, 52 (7.7%) of the samples had preterm labor. In the study of the status of intermediary social determinants of health, 231 (34.1%) subjects had food insecurity, and 76.4%, 85.5%, and 86.2% of the cases did not experience stress, anxiety, and depression, respectively. As regards social support, 15.4%, 34.2%, and 50.4% of the participants had low, moderate, and high social support. Further, 302 (44.8%) of the participants had experienced domestic violence, and 89 (13.2%) of the pregnancies were unwanted. 

No significant association was noted between the structural determinants of health and preterm delivery, women's education (p=0.58), husband's education (p=0.14), women's occupation (p=0.40), household size (p=0.11), household income (p=0.70), and household costs (p=0.56) in women with term and preterm delivery (p>0.05). Only a significant association was observed between the husband's job and preterm labor (p=0.007). The results of logistic regression analysis reflected that the probability of premature delivery in those with self-employed husbands was 70%, and employed husbands were 87% less than those with unemployed spouses (Table II). 

In examining the relationship between intermediary determinants of health and preterm labor, a significant association was found between preterm delivery and food insecurity (p=0.015), stress (p=0.027), and inadequate prenatal care (p≤0.001). Logistic regression analysis demonstrated that the likelihood of preterm labor in subjects with food insecurity, stress, and inadequate care during pregnancy was 2, 9.1, and 13.2 times higher than those with food safety, less stress, and adequate care (Table III).

**Table I:** The demographic and socio-economic characteristics of the participants

**Variable**		**Mean ± SD**	**Min-Max**
Women’s age*		28.59 ± 4.42	18-35
Husband’s age*		33.25 ± 5.43	21-53
Number of years of women education*		13.03 ± 3.28	4-22
Number of years of husband education*		13.00 ± 3.35	2-22
Numbers of pregnancy*		1.77 ± 0.92	1-7
Women’s occupation**			
	Housewives	582 (86.4)	
	Employees	92 (13.6)	
Husband’s occupation**			
	Unemployed	12 (1.8)	
	Employees	233 (34.7)	
	self-employed	429 (63.5)	
Family size**			
	1-3	527 (78.2)	
	4 and above	147 (21.8)	
Household’s income**			
	< 10 million rials	334 (49.6)	
	≥10 million rials	340 (50.4)	
The average household costs**			
	< 10 million rials	116 (17.2)	
	≥10 million rials	558 (82.8)	

* Data presented as mean±SD.

** Data presented as n (%)

**Table II:** The relationship between socio-economic characteristics of the participants with preterm birth

**Variable**		**Preterm**	**Term**	**OR (CI: 95%)**	**p-value**
Women's education					
	Secondary	7 (10.1)	62 (89.9)	Ref	0.588
	Middle	17 (6.6)	240 (93.4)	0.62 (0.24-1.58)	
	High school	28 (8.0)	320 (92.0)	0.77 (0.32-1.85)	
Husband's education					
	Secondary	9 (13.2)	59 (86.8)	Ref	0.146
	Middle	22 (8.0)	252 (92.0)	0.57 (0.25-1.30)	
	High school	21 (6.3)	311 (93.7)	0.19 (0.19-1.04)	
Women's occupation					
	Housewives	43 (7.4)	539 (92.6)	Ref	0.403
	Employees	9 (9.8)	83 (90.2)	1.35 (0.63-2.89)	
Husband's occupation					
	Unemployed	3 (25.0)	9 (75.0)	Ref	0.007
	Employees	39 (9.1)	390 (90.9)	0.30 (0.07-1.15)	
	Self-employed	10 (4.3)	223 (95.7)	0.13 (0.03-0.57)	
Household size					
	1-3	36 (6.8)	491 (93.2)	Ref	0.116
	4 and above	16 (10.9)	131 (89.1)	1.66 (0.89-3.09)	
Household income					
	<10 million Rials	10 (8.6)	106 (91.4)	Ref	0.702
	≥10 million Rials	42 (7.5)	516 (92.5)	0.86 (0.42-1.77)	
The average household costs					
	<10 million Rials	0.28 (8.4)	306 (91.6)	Ref	0.565
	≥10 million Rials	24 (7.1)	316 (92.9)	0.83 (0.47-1.46)	

Data presented as n (%).

P-value <0.05 was considered significant; and the data were analyzed using Chi-square test, Fisher's exact test and logistic regression

**Table III:** The relationship between intermediate social determinants of health with Preterm birth

**Variable**		**Preterm**	**Term**	**OR (CI: 95%)**	**p-value**
Food security					
	Food security	26 (5.9)	417 (94.1)	Ref	0.015
	Food insecurity	26 (11.3)	205 (88.7)	2.03 (1.15-3.59)	
Social support					
	Low	10 (9.7)	93 (90.3)	Ref	0.687
	Moderate	26 (7.6)	316 (92.4)	0.76 (0.35-1.64)	
	High	16 (7.0)	213 (93.0)	0.69 (0.30-1.59)	
Stress					
	No	33 (6.4)	482 (93.6)	Ref	0.027
	Yes	19 (11.9)	140 (88.1)	1.98 (1.09-3.59)	
Anxiety					
	No	42 (7.3)	534 (92.7)	Ref	0.007
	Yes	10 (10.2)	88 (89.8)	1.44 (0.69-2.98)	
Depression					
	No	41 (7.1)	540 (92.9)	Ref	0.139
	Yes	11 (11.8)	82 (88.2)	1.76 (0.87-3.57)	
Pregnancy's worries and stress					
	No	33 (6.7)	463 (93.3)	Ref	0.101
	Yes	19 (10.7)	159 (89.3)	1.67 (0.92-3.03)	
Domestic violence					
	No	30 (9.9)	272 (90.1)	Ref	0.059
	Yes	22 (5.9)	350 (94.1)	1.75 (0.99-3.11)	
Unwanted pregnancy					
	No	44 (7.5)	541 (92.5)	Ref	0.669
	Yes	8 (9.0)	81 (91.0)	1.21 (0.55-2.67)	
Prenatal care					
	Adequate	11 (2.2)	486 (97.8)	Ref	<0.001
	Inadequate	41 (23.2)	136 (76.8)	13.32 (6.66-26.61)	

Data presented as n (%).

P-value <0.05 was considered significant; and the data were analyzed using Chi-square test, Fisher's exact test and logistic regression

## Discussion

The prevalence of preterm delivery was 7.7% in the present study. Its prevalence varies across different parts of the world. However, in 2010, the WHO reported that a total of 135 live births occur all over the world, 14.9 million (11.1%) of which are premature; South Africa and Asia constitute 60% of cases of premature delivery (17). According to former studies, the highest rates were of preterm delivery reported from low-income countries (11.8%), followed by middle-income (11.3%) and high-income (9.3%) countries (18). The reason for this discrepancy could be different study methods, study populations, and incongruous definitions for premature birth. 

The present study found no significant relationship between socioeconomic factors and preterm labor. Kramer and colleagues believed that unfavorable economic condition is unlikely to be a direct and independent factor for preterm delivery. However, it can cause premature delivery through unhealthy behaviors, exposure to stress, and psychological responses to stress (19). In a meta-analysis by Sharifi and colleagues, no significant relationship between socioeconomic factors and preterm delivery was pointed out (2). Socioeconomic status is one of the key determinants of health and mortality that can affect pregnancy outcomes. Families with low socioeconomic status suffering from problems such as malnutrition, inadequate prenatal care, addiction, smoking, alcohol consumption, frequent pregnancies, and stress are prone to undesirable pregnancy outcomes (20).

Incongruent findings can be due to different categorizations of socioeconomic factors, which highlight the need for performing further studies with similar methods and identical standardized questionnaires for assessing the socioeconomic status to enhance comparability of the results Mothers' mental health during pregnancy affects maternal and fetal development. According to the recent evidence, there is a relationship between pregnancy outcome and stressful life events, anxiety, depression, stressful occupation, physical abuse, and low social support (21). Among the psychosocial factors, we only found a linkage between stress and preterm labor. The probability of preterm delivery in stressed individuals was higher than those not experiencing stress. Nonetheless, no significant relationship was found between preterm labor and other factors such as social support, anxiety, depression, violence, and unwanted pregnancy. Researchers have identified pregnancy anxiety as a strong predictor of premature delivery, and depression and chronic stress as more powerful predictors of birth weight (10). Considering that psychological factors such as stress, anxiety, and depression are not evaluated in the routine prenatal care, the level of these problems during pregnancy is unclear and their effects on maternal and fetal health remain to be inconspicuous. Therefore, to improve maternal health and prevent adverse maternal and neonatal outcomes, evaluating maternal psychological factors in each trimester of pregnancy as a part of the routine care is essential.

Food insecurity represents a major public health concern and a useful indicator of health and well-being, which is considered as one of the effective social determinants of health (22). In the current study, there was a significant relationship between food insecurity and preterm labor. The odds of preterm delivery were higher in patients with food insecurity. To the best of our knowledge, no studies have been conducted evaluating the association of food insecurity with preterm labor. The results of other studies pinpointed the association of food insecurity with maternal adverse outcomes such as gestational diabetes, excessive overweight during pregnancy, and hypertension (11, 23).

Considering the scarcity of studies on food insecurity during pregnancy and its association with adverse pregnancy outcomes, the present study can be used as a foundation for future studies. Comparison of our obtained results with those of future studies would allow implementing beneficial interventions for this important population group and promoting maternal and neonatal health status. Prenatal care is also one of the social determinants of health. In the present study, we observed a significant relationship between inadequate prenatal care and preterm delivery. In a study by Alizadeh and colleagues, patients with inadequate prenatal care were 1.51 times more likely to be exposed to premature delivery (24). Likewise, Vintzileos and colleagues revealed that the prevalence of premature labor was 2.8 higher in patients with inadequate prenatal care (25).

A review of various studies exhibited that participation in maternity and prenatal classes can assuage anxiety due to receiving information and care and using the experiences of other mothers, which can play a key role in reducing diseases, their complications, and improving health through increased knowledge and skills (26). The strength of the present study was considering several social determinants of health. The longitudinal nature of the study is also another strength of the study because many studies in this area had a cross-sectional design. In addition, food insecurity during pregnancy is one of the most important factors that has been considered in a limited number of studies. The present results can set the path for future studies.

## Conclusion

Social determinants of health are important factors affecting pregnancy outcomes, among which intermediary factors of social determinants of health are the most critical factors affecting the adverse outcomes of pregnancy such as preterm labor. Therefore, to reduce the adverse outcomes of pregnancy, performing interventions on these factors seems to be mandatory.
